# Combined optical coherence tomography and electroretinography system for imaging neurovascular coupling in the human retina

**DOI:** 10.1117/1.NPh.12.3.035004

**Published:** 2025-08-09

**Authors:** Khushmeet Dhaliwal, Alexander Wong, Tom Wright, Kostadinka Bizheva

**Affiliations:** a University of Waterloo, Department of Physics and Astronomy, Waterloo, Ontario, Canada; b University of Waterloo, Systems Design Engineering Department, Waterloo, Ontario, Canada; c Kensington Vision and Research Center, Toronto, Ontario, Canada; d University of Toronto, Department of Ophthalmology and Vision Science, Toronto, Ontario, Canada; e University of Waterloo, School of Optometry and Vision Sciences, Waterloo, Ontario, Canada

**Keywords:** optical coherence tomography, functional imaging, retinal imaging, human retina, neurovascular coupling

## Abstract

**Significance:**

During their early stages of development, neurological and neurodegenerative diseases cause changes to the biological tissue’s morphology, physiology and metabolism at the cellular level, and acute, transient changes in the local blood flow. The development of optical methods that can image and quantify such changes simultaneously and investigate the relationship among them (neurovascular coupling) in neural tissues can have a profound effect on furthering our understanding of neurodegeneration.

**Aim:**

Our aim is to develop an optical imaging platform for imaging and characterization of neurovascular coupling in the human retina with high spatial and temporal resolutions.

**Approach:**

A compact, clinically viable optical coherence tomography technology was developed for *in vivo*, simultaneous structural, functional, and vascular imaging of the human retina and was integrated with a clinical electroretinography system. Image processing algorithms were developed to measure visually evoked physiological and blood flow changes in the living retina and explore neurovascular coupling in the healthy human retina.

**Results:**

Both intensity and optical path length changes were measured with optical coherence tomography from most major retinal layers (nerve fiber layer, plexiform layers, inner and outer segments of the photoreceptors, and the retinal pigmented epithelium) in response to a visual stimulation with a 4-ms single white light flash. The visual stimulus also caused fast transient changes in the retinal blood flow in the local blood vessels. The time courses of these changes were similar, and their magnitude was proportional to the intensity of the visual stimulus.

**Conclusions:**

We have developed an optical imaging modality for non-invasive probing of neurovascular coupling in the living human retina and demonstrated its utility and clinical potential in a pilot study on healthy subjects. This imaging platform could serve as a useful clinical research tool for investigation of potentially blinding retinal diseases, as well as neurodegenerative brain diseases that are expressed in the retina such as Alzheimer’s and Parkinson’s diseases.

## 1 Introduction

The retina, often called “the window to the brain,” is an extension of the central nervous system (CNS) designed for the detection of visual information and its transmission to the visual cortex of the brain.^1^ Highly specialized retinal neurons, such as photoreceptors which detect light and filter it by spectral content; bipolar and horizontal cells which filter the photoreceptor responses spatially and temporally; and ganglion cells that amplify the visual information and transmit it to the brain, are organized in well-defined layers.^2^^–^^5^ Furthermore, the retina is highly vascularized with oxygen and nutrition delivered to the retinal neurons in the anterior retina by the inner retina vasculature and to photoreceptors by the choroidal vasculature.^6^^,^^7^ Neurovascular coupling in the retina is referred to as a temporary increase in the retinal blood flow and vasodilation of the retinal blood vessels in response to the increased metabolic demand of retinal neurons. This phenomenon was first observed in the brain^8^ and later in the retina^9^^,^^10^ using non-invasive optical imaging methods.

Potentially blinding retinal diseases such as age-related macular degeneration,^11^^,^^12^ diabetic retinopathy,^13^^–^^15^ retinitis pigmentosa,^16^^–^^18^ and glaucoma^19^^–^^21^ affect a large portion of the world’s aging population and amount to >$1.5B in direct costs to the Canadian Health Care System annually.^22^ These diseases cause progressive loss of normal function and eventually irreversible cell death of retinal neurons that, in the later stages of disease development, are registered as morphological changes. As these diseases cause metabolic and physiological changes in the retinal neurons, studies have reported blood flow changes with their progression.^23^^,^^24^ However, the dynamic relationship between neuronal and blood flow changes (neurovascular coupling) is still not fully understood in the healthy and diseased human and animal retina partly due to the fact that, in the past, functional and vascular studies have been conducted separately.

Optical coherence tomography (OCT) is an imaging modality that can generate non-invasively volumetric images of the retina with micrometer-scale resolution, sufficient for visualization of the fine layered structure of the human retina.^25^^–^^29^ When combined with adaptive optics, OCT is also able to visualize individual retinal neurons.^30^^–^^32^ Doppler OCT can be used to measure retinal blood flow,^33^^–^^35^ whereas optical microangiography (OMAG) can be used to map the retinal vasculature in 3D over a wide field of view.^36^^–^^38^ All of these OCT-based technologies have found valuable applications in ophthalmology for clinical research of various ophthalmic diseases.^18^^,^^37^^,^^39^^,^^40^ Both Doppler OCT^41^^–^^43^ and OMAG^44^^,^^45^ technologies have been used in the recent past to evaluate blood flow changes in the human retina evoked by visual stimulation. However, these studies utilized very long stimuli (>5  s) and did not record the retinal blood flow (RBF) changes continuously.^41^^,^^43^^,^^46^^,^^47^ Therefore, they were not able to provide information about the transient behavior of the RBF with high temporal resolution.

Optoretinography (ORG) is the optical equivalent of electroretinography (ERG), which is a clinically established method for electrical recordings of the responses of retinal neurons to visual stimulation. The use of OCT for non-invasive, optical recording of visually evoked neuronal changes was first proposed by Bizheva et al.,^48^ and the proof-of-principle was demonstrated *in vitro* in animal tissue where OCT and ERG data were acquired synchronously. With advances in camera and laser technologies, over the past 20 years, different OCT system designs were developed for ORG recordings of visually evoked neuronal activity in the human and animal retina: point scanning spectral domain OCT,^49^ AO-OCT,^30^^,^^31^ full-field SS-OCT,^50^^–^^53^ and line-field SD-OCT.^54^^,^^55^ Although the majority of the ORG data were acquired from healthy retinas, most recent studies have explored the changes in the ORG signal related to retinal diseases.^18^

Here, we present the design of a novel, combined OCT+ERG system, imaging protocols, and image processing algorithms that were developed for simultaneous probing of visually evoked changes in the human retina neuronal function and RBF with high spatial and high temporal resolutions. The OCT+ERG imaging modality can serve as a new research tool for exploring neurovascular coupling in the human retina.

## 2 Methods

The following paragraphs describe the optical design of the OCT system, the imaging protocols for the data collection, and the image processing algorithms for analysis of the neuronal and vascular responses of the living retina to visual stimulation.

### 2.1 OCT+ERG System

**Fig. 1:**
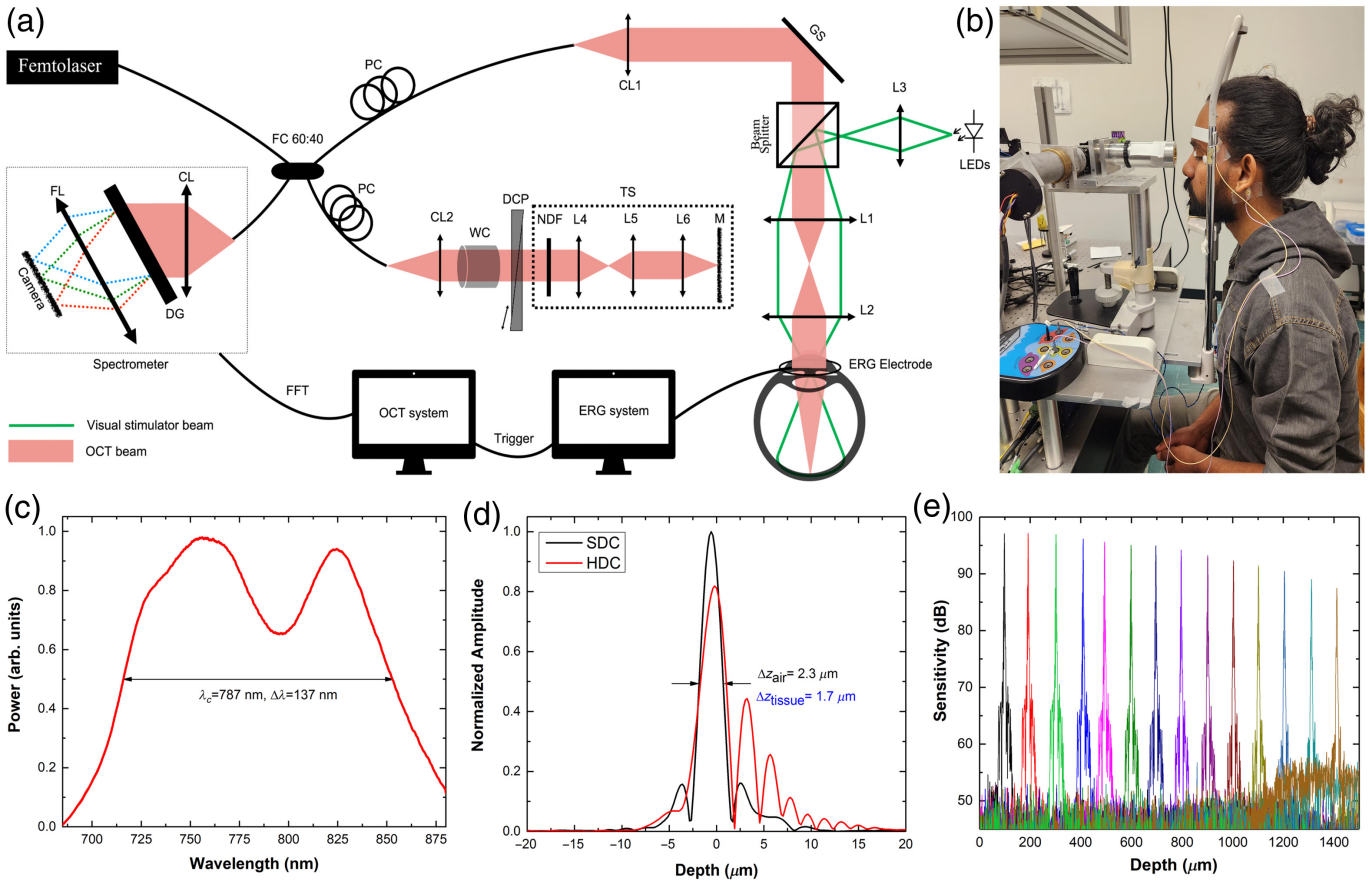
(a) Schematic of the combined OCT+ERG system. CL, collimator lens; DCP, dispersion compensation prism; DG, diffraction grating; FC, fiber coupler; FFT, fast Fourier transform; FL, focusing lens; GS, galvanometric scanner; L1–L6, achromatic doublet lenses; M, reference mirror; NDF, neutral density filter; PC, polarization controller; TS, translation stage; WC, water cell. (b) Photograph of the OCT+ERG imaging probe. (c) Laser spectrum measured from the reference arm of the OCT system. (d) Axial OCT PSF after hardware (red color) and software (black color) dispersion compensation. (e) Sensitivity roll-off with imaging depth.

A combined OCT+ERG system was developed for non-invasive probing of neurovascular coupling in the living human retina. The optical design of the compact, fiber-optic, spectral-domain OCT system is shown in Fig. 1, whereas a photograph of the OCT imaging probe integrated with a custom visual stimulator is presented in Fig. 1(b). The output of a femtosecond laser (Femtolasers GmbH, Vienna, Austria) was connected to a spool of a 100-m-long fiber to stretch the femtosecond pulses and mimic continuous wave (CW) emission over a broad spectral range (∼670 to 920 nm). The output from the spool was interfaced with a fiber-optic coupler (60/40 split ratio, Gould Fiberoptics, Millersville, Maryland, United States) that served as the core of the OCT interferometer.

In the imaging arm of the OCT system, a parallel optical beam of 2.8 mm diameter was generated with a fiber collimator (CL1—achromat doublet; f=10  mm, Thorlabs, Newton, New Jersey, United States). A pair of galvanometric scanners (Cambridge Technologies, Worthington, Minnesota, United States) was used to scan the imaging beam over the retinal surface. A telescope comprising two achromat doublet lenses [L1 (f=75  mm) and L2 (f=60  mm), Thorlabs] was used to generate a parallel optical beam of 2.24 mm diameter and an optical power of ∼1.1  mW that was projected onto the human cornea. The mechanical design of the telescope allowed for easy adjustment of the distance between lenses L1 and L2 to compensate for myopia and hyperopia in the imaged subjects and fine-tune the focusing of the imaging beam within the retina. A custom visual stimulator comprising a wheel with four light emitting diodes (LEDs) (white, blue, green, and red) was integrated with the OCT imaging probe through lens L3 (f=50  mm, Thorlabs) and a pellicle beam splitter (T/R ratio of 92/8, Thorlabs). This design allows for the light from one of the LEDs to be focused at the pupil plane of the human eye to generate a wide field of view illumination of the retina with almost uniform intensity (Maxwellian view). The custom visual stimulator was interfaced with a commercial ERG system (Espion 2, Diagnosys LLC, Lowell, Massachusetts, United States), which served two purposes: (a) precise control of the timing, duration, intensity and frequency of visual stimulus and (b) simultaneous collection of ERG and OCT recordings, so that the ERG recordings are used as a “gold standard” to validate the ORG traces extracted from the OCT images.

The reference arm of the OCT interferometer comprised a fiber collimator (achromat doublet, f=10  mm, Thorlabs), a custom-built 1-cm-long water cell (WC) for crude compensation of water dispersion of the OCT imaging beam within the human eye, a custom dispersion compensation (DCP) unit comprising BK7 prisms mounted on miniature translation stages (Edmund Optics, Barrington, New Jersey, United States), a telescope [L4 and L5, achromat doublets (f4=f5=60  mm), Thorlabs], a focusing lens [L6, achromat doublet (f=10  mm), Thorlabs], and a mirror mounted on a miniature translation stage (Edmund Optics). The telescope and the focusing mirror were mounted on a manual translation stage (Edmund Optics). Polarization controllers (PCs) were used in both arms of the interferometer.

The detection end of the OCT system comprised a commercially available spectrometer (Cobra-S 800, Wasatch Photonics, Durham, North Carolina, United States), interfaced with a line-scan CMOS camera (OCTOPLUS, Teledyne, Thousand Oaks, California, United States) with a readout rate of 250 kHz.

Figure 1(c) shows the spectrum of the optical beam measured at the detection end of the OCT system, which accounts for spectral changes induced by the optical transmission function of the OCT system. Figure 1(d) shows the axial point spread function (PSF) after hardware dispersion compensation only (red line) and after additional software dispersion compensation (black line), based on previous publications.^56^ The OCT system’s maximum sensitivity measured for 1.1-mW imaging power, which is well below the maximum permissible exposure as specified by the ANSI standard^57^ and camera speed of 250 kHz was 98 dB with a roll-off of ∼10  dB over a scanning range of 1.5 mm [Fig. 1(e)].

### 2.2 In Vivo Imaging of the Human Retina

A pilot study to test the clinical viability of the OCT+ERG system was conducted on two healthy subjects (two males, age range 25 years ± 5 years). The study received full ethics clearance from the University of Waterloo’s Office of Research Ethics, and a written consent was obtained from all participants. The study participants underwent standard clinical examination prior to the OCT imaging sessions, and only subjects with no ocular diseases or a history of epilepsy and nearly perfect vision (minor correction up to −1 diopters) were included in the study. A Dawson, Trick, and Litzkow (DTL)-type ERG electrode was inserted under the lower eyelid of the imaged eye, whereas reference and ground ERG electrodes were applied to the participant’s temple and behind the ear, respectively [Fig. 1(b)]. Both pupils were dilated by application of one drop of tropicamide in each eye followed by ∼30-min dark adaptation. A faint red LED was used as a fixation target for the non-imaged eye.

### 2.3 Visual Stimulation and Image Acquisition Protocols

**Fig. 2:**
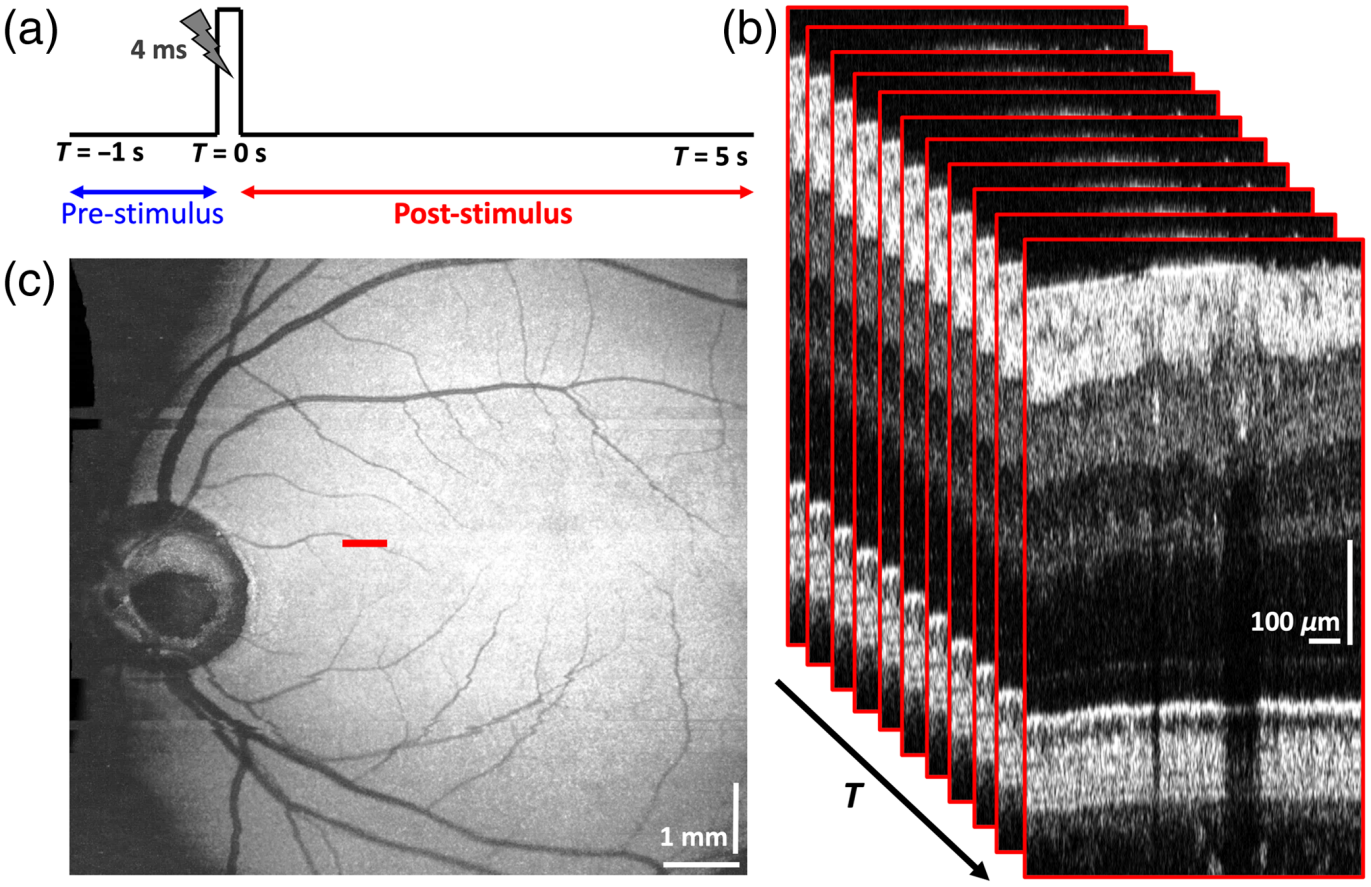
Image acquisition protocol for the optoretinogrpahy and functional blood flow recordings. (a) Timeline of the visual stimulus. (b) Sequence of 1200 repeated B-scans acquired over a duration of ∼6  s from a location in the retina approximately midway between the fovea and optic nerve head (c, red line).

In this study, the retina was stimulated with white-light single flashes of 4-ms duration and different intensities ranging from $0\;\mathrm{cd.}s/m^{2}$ (dark recordings) to $43.4\;\mathrm{cd.}s/m^{2}$. OCT and ERG data were acquired over a period of ∼6  s (1 s pre-stimulus and 5 s post-stimulus), as shown in Fig. 2(a), to minimize the effect of ocular motion on the ORG data. Table 1 shows the percentage of bleached photoreceptors for each stimulus intensity used in this study, calculated based on the method described by Rushton and Henry.^58^^,^^59^

**Table 1:** Percentage of bleached photoreceptors for different stimulus intensities.

Stimulus intensity ($\mathrm{cd.}s/m^{2}$)	Bleaching (%)
1.4	1.4
14.5	13.8
43.4	36

Large field of view, volumetric morphological OCT images of the retina (1000 A-scans × 1000 B-scans) were acquired prior to the acquisition of the functional OCT recordings to map the vasculature on the retinal surface. Next, a series of 1200 repeated OCT B-scans, each composed of 1000 A-scans [Fig. 2(b)], were acquired from a location in the retina positioned ∼10  deg from the fovea [red line in Fig. 2(c)]. Although our intention was to acquire the repeated B-scans from the same location, involuntary ocular motion can shift the imaging beam slightly along the *en face* plane. As in our study we are examining the group response of all retinal photoreceptors by averaging the ORG data along the width of each B-scan, rather than recording ORG traces from individual photoreceptor as measured with AO-OCT,^30^^,^^55^ the effect of ocular microsaccades on the ORG data is suppressed significantly.

### 2.4 Image Processing and Data Analysis

**Fig. 3:**
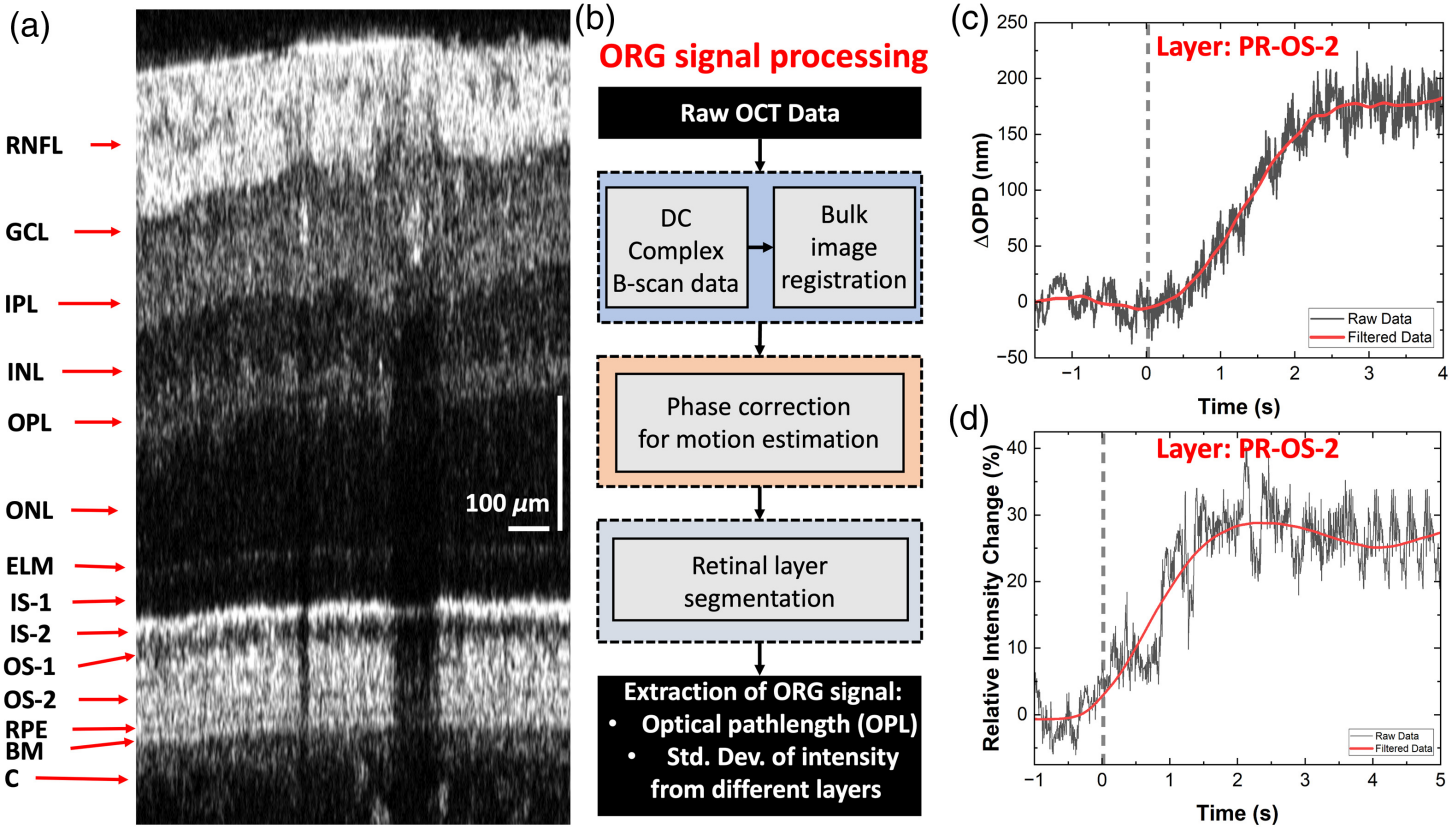
Protocol for extracting ORG traces from the repetitive B-scans. (a) Representative B-scan (no averaging) showing the layered structure of the human retina and cross-sections of the large blood vessels in the RNFL. RNFL, retinal nerve fiber layer; GCL, ganglion cell layer; IPL, inner plexiform layer; INL, inner nuclear layer; OPL, outer plexiform layer; ONL, outer nuclear layer; ELM, external limiting membrane; IS-1, inner segment 1; IS-2, inner segment 2; OS-1, outer segment 1; OS-2, outer segment 2; RPE, retinal pigment epithelium; BM, Bruch’s membrane; C, choroid. (b) Flow chart of the image processing steps to extract the OPD and intensity ORG traces from different retinal layers. Example ORG traces: OPD (c) and intensity (d) extracted from the OS-2 band of the PR layer.

Custom MATLAB-based algorithms were developed for processing and analysis of the OCT data. The raw OCT data were first processed to generate dispersion-compensated complex-valued B-scans [representative intensity image shown in Fig. 3(a)]. Figure 3(b) shows a flow chart for the image processing steps designed to measure ORG responses (optical path length and intensity changes) from different retinal layers. To account for involuntary eye motion caused by breathing, heartbeat, and eye muscle twitching, a sub-pixel phase-restoring image registration method was utilized to correct for motion-related misalignment among consecutive B-scans.^60^^–^^62^ Next, a region of interest (ROI) located outside the shadows of the retinal blood vessels and visible throughout the time series was defined for each B-scan of the time series, the retinal layers were segmented automatically based on their intensity profiles using boundary detection,^63^ and the B-scans were flattened using the photoreceptors IS-2 layer as a guideline. The segmented data were used to extract optical path length and intensity changes for each retinal layer in response to visual stimulation. To generate the intensity traces, the values for all pixels within each segmented layer were averaged axially as well as laterally, and standardized intensity change was measured as described previously by Cooper et al.^64^^,^^65^ Optical path length changes within the segmented layers were calculated based on the following equation:

$$\Delta \mathrm{OPD}=\frac{\lambda_{0}}{4\pi }\times (\phi_{\text{target}}-\phi_{\text{reference}}),$$ (1)

where ΔOPD represents the optical path length changes between the reference and the target layers averaged in the lateral as well as the axial directions, $\lambda_{0}$ is the central wavelength of the imaging light source, and $\left(\phi_{\text{target}}-\phi_{\text{reference}}\right)$ represents the phase difference between the target retinal layer and the reference layer. The inner limiting membrane was selected as the reference layer for all the inner retinal target layers, and IS-2 was selected as the reference layer for measuring ORG changes in the photoreceptor (PR) OS-2.

Representative optical path length difference (OPD) and intensity traces extracted from the photoreceptor OS-2 layer are shown in Figs. 3(c) and 3(d), respectively. The high-frequency data (black line) resulting from the OCT speckle pattern and periodic modulations related to breathing and heart rate were filtered out using a combination of filtering functions to generate the smooth time traces (red curves) in Figs. 3(c) and 3(d).

**Fig. 4:**
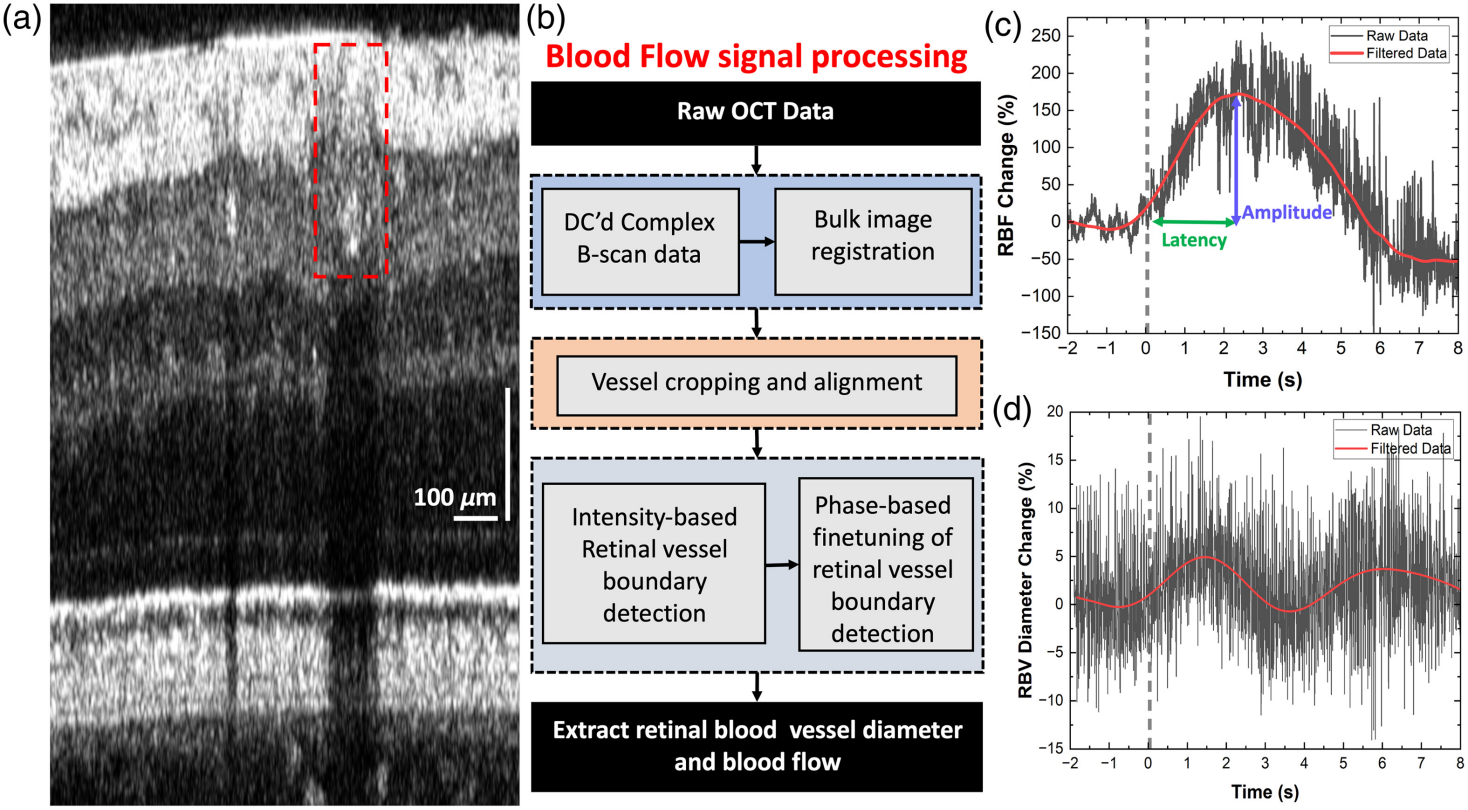
Protocol for measuring transient changes in the retinal blood vessels. (a) Representative retinal B-scan with the cross-section of a retinal blood vessel marked with the red dashed line. (b) Flow chart of the image processing steps to quantify dynamic vascular responses (blood flow change and dilation/constriction of retinal blood vessels) to visual stimulation. (c) Retinal blood flow change in response to 4-ms single flash white-light stimulus. (d) Corresponding blood vessel diameter change.

A custom algorithm was also developed for quantifying vascular changes (retinal blood flow and blood vessel diameter) caused by visual stimulation. Figure 4(a) shows a representative B-scan, where an ROI including the cross-section of a retinal blood vessel is marked with the red dashed line. Figure 4(b) shows a flow chart of the algorithm developed for analysis of the vascular changes.

Bulk registration was used to align all B-scans for eye motion.^60^ Next, a blood vessel was manually selected from the first B-scan in the time series by looking at the shadow cast by the vessel in the deeper layers of the retina, as blood cells are highly reflective. The width of the shadow was correlated to the diameter of the blood vessel as the lumen of retinal blood vessels has much lower reflectivity. The blood vessel was cropped from all B-scans in the time series using a semi-automated algorithm. These data were used to extract the blood velocity information based on the phase shift among the adjacent A-scans.^33^^,^^66^ The RBF was obtained from each B-scan using

$$F=V\times \frac{\pi D^{2}}{4},$$ (2)

where F is the retinal blood flow, V is the retinal blood velocity, and D is the diameter of the blood vessel cross-section. Therefore, the blood velocity and vessel area information (in appropriate units) were used to evaluate visually evoked RBF changes over the time series of B-scans.

Representative RBF and blood vessel diameter (BVD) time traces are shown in Figs. 4(c) and 4(d). The raw data exhibited high fluctuations (black lines) because of the speckle present in the OCT images. A second-order polynomial smoothing filter with a 199-point moving window (Savitzky–Golay) was applied twice to filter out the high-frequency component of the data. Next, the filtered data were zero-padded (∼3×) to enable band-block filtering of the pulsatile fluctuations (0.37 to 1.3 Hz) caused by the participant’s heart rate and to generate smooth traces (red lines) of the time-dependent changes of the RBF and BVD caused by the visual stimulation of the retina. Fractional changes in the RBF were computed based on the following relation:

$$\mathrm{RBF}\;\text{Change}\;(\%)=\frac{\text{Signal}\;\mathrm{RBF}\;\text{value}\;(A)-\text{Baseline}\;\mathrm{RBF}\;\text{value}\;(B)}{\text{Baseline}\;\mathrm{RBF}\;\text{value}\;(B)}\times 100,$$ (3)

where the signal RBF value corresponds to the RBF value obtained for a vessel in each B-scan, and the baseline RBF value corresponds to the mean RBF value during the pre-stimulus period. A similar analysis was conducted to compute fractional changes for BVD response in the axial and lateral directions

$$\mathrm{BVD}\;\text{Change}\;(\%)=\frac{\text{Signal}\;\mathrm{BVD}\;\text{value}\;(A)-\text{Baseline}\;\mathrm{BV}D\;\text{value}\;(B)}{\text{Baseline}\;\mathrm{BVD}\;\text{value}\;(B)}\times 100.$$ (4)

Here, the signal BVD value represents the value of the cross-sectional diameter of a blood vessel in a B-scan, and the baseline BVD value is determined by taking the average of the vessel diameter fluctuations over pre-stimulus recording.

To quantify the RBF and BVD response to visual stimulation, we employed two metrics: “amplitude,” measured relative to the baseline (pre-stimulus data), and “latency,” measured from the onset of the visual stimulus to the time of the peak response [Fig. 4(c)].

## 3 Results

**Fig. 5:**
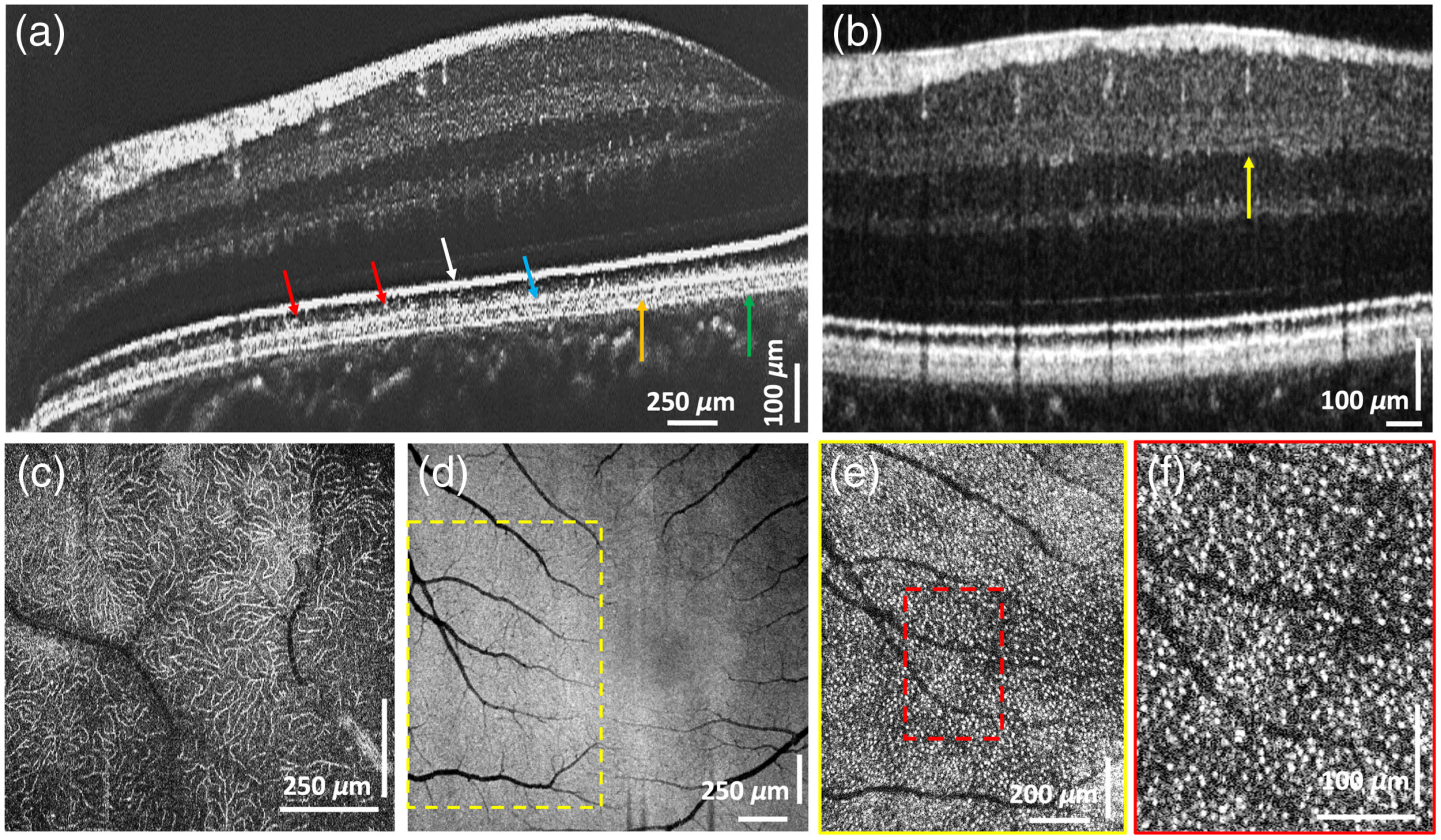
OCT images of the healthy human retina. The high spatial resolution is sufficient to resolve the Bruch’s membrane (green arrow), the RPE (orange arrow), the photoreceptors OS-2 layer (blue arrow) and reflections from individual cone OS tips (red arrow) (a), the three sub-layers in the retinal IPL (b), and the intricate capillary network in the retinal OPL (c). *En face* view of the photoreceptor IS/OS junction (d). Magnified views of the photoreceptor mosaic, where the tips of individual cones are resolved [white dots in panels (e) and (f)].

Figure 5 shows the representative cross-sections and *en face* morphological images of the healthy human retina. In Fig. 5(a), 10 B-scans were averaged with 5000 A-scans per B-scan, scanning over ∼4.5-mm region. The high axial OCT resolution allowed for clear visualization of the Bruch’s membrane [Fig. 5(a), green arrow]; the retinal pigmented epithelium [RPE, Fig. 5(a), orange arrow]; the posterior end of the photoreceptors outer segment [OS-2, Fig. 5(a), blue arrow] that contains RPE micro-villi rich in melanin granules, responsible for the high reflectivity of this sub-layer; reflections from cone tips in the anterior part of the photoreceptor’s outer segment [Fig. 5(a), red arrows]; and the highly reflective posterior end of the photoreceptor’s inner segment [IS-2, Fig. 5(a), white arrow] that contains densely packed mitochondria. The high axial resolution also allows for visualization of three distinct sub-bands in the retinal inner plexiform layer [IPL, Fig. 5(b), yellow arrow] that were previously observed with research-grade visible light OCT technology with comparable axial resolution.^27^

Figure 5(c) shows an *en face* view of the microvasculature at the outer plexiform layer (OPL) of the retina. Figure 5(d) shows an *en face*, maximum intensity projection image of the photoreceptors IS/OS junction. Figures 5(e) and 5(f) show the magnified views of an ROI located a few degrees away from the center of the fovea, where reflections from cones can be observed (highly reflective white dots).

**Fig. 6:**
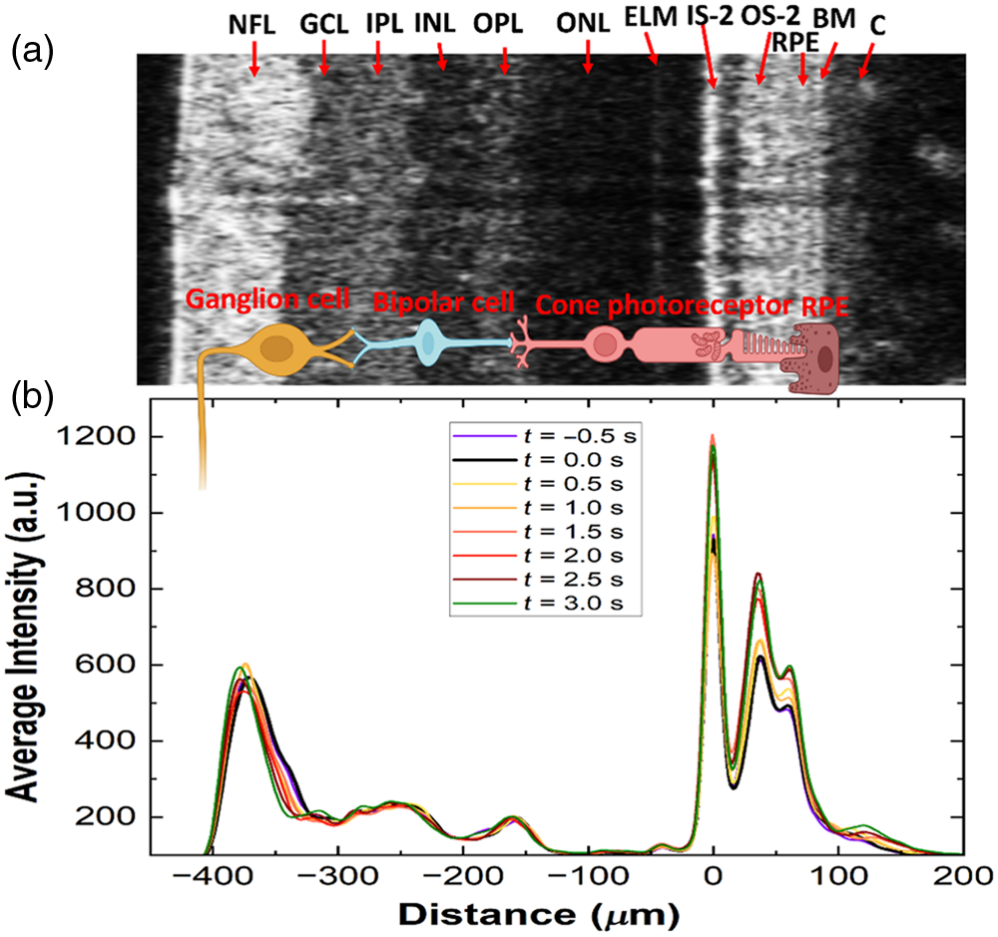
(a) Representative B-scan of the healthy human retina from a functional OCT time series with layers labeled. (b) Retina intensity profiles extracted from the series of B-scans for selected time points before, during, and after application of the visual stimulus. Pictorial representation of the cellular pathway for the generation of visual information.

Figure 6(b) shows the intensity profiles of the human retina generated from a time series of B-scans [a single representative B-scan from the series is shown in Fig. 6(a)] for selected time points before, during, and after the single flash stimulus. A flat region in each B-scan was chosen to average the intensity values across the B-scan. The intensity profiles were aligned with respect to each other, using the peak corresponding to the photoreceptor IS-2 layer as a reference point. A simplified pictorial representation of the retina’s cellular pathway for generation and transmission of visional information is also shown in Fig. 6(a). The figure shows clearly that visual stimulation causes time-dependent increases and decreases in the intensity of different retinal layers, as well as temporary swelling in some of the retinal layers, which results in spatial shifts of the intensity peaks corresponding to those retinal layers (optical path length changes).

**Fig. 7:**
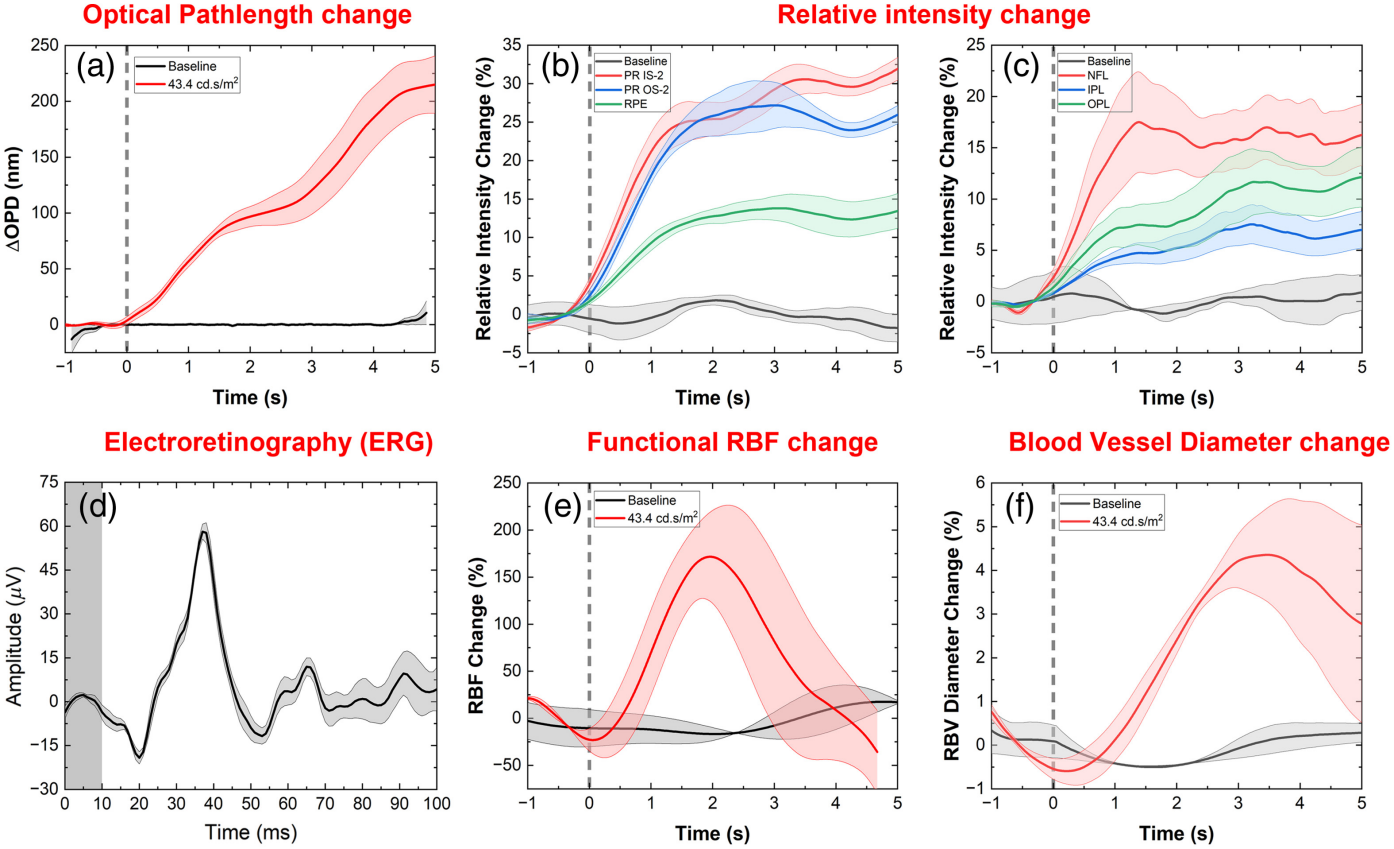
Summary of the healthy human retina response to visual stimulation (white light, single flash, 4-ms duration, and $43.4\;\mathrm{cd.}s/m^{2}$ intensity) in terms of physiological changes in the retinal neurons measured with OCT (intensity and OPD changes) and ERG, and vascular changes (transient changes in the RBF and BVD). The block line represents an average of at least three measurements from one subject, and the shaded area represents the standard error of the measurements. (a) Optical path length changes from the PR OS-2 layer. Intensity changes measured from the retinal layers located in the posterior (b) and anterior (c) retinas. (d) Representative ERG recording. Visually evoked changes in the retinal blood flow (e) and blood vessel diameter (f).

Figure 7 shows a summary of the retinal response to visual stimulation (4-ms duration and $43.4\text{-}\mathrm{cd.}s/m^{2}$ intensity) in terms of physiological changes in the retinal neurons measured with OCT (intensity and OPD changes) and ERG and vascular changes (transient changes in the RBF and BVD). Figure 7(a) shows phase-based optical path length changes in the photoreceptors OS-2 band (red line). The photoreceptors show an expansion in two steps, reaching a value of ∼220  nm at ∼5  s after the stimulus onset. The black line shows the OPD changes during baseline (dark recording).

Figures 7(b) and 7(c) show standardized intensity-based changes in the retinal layers. Figure 7(b) shows the intensity changes in the outer retina, specifically the IS-2 (red curve), the OS-2 (blue curve), and the RPE (green curve). The intensity changes observed in the IS-2 and the OS-2 layers are similar in magnitude (∼30%); however, the peak intensity change for the OS-2 layer occurs a few milliseconds earlier than the change in the IS-2 layer. The intensity change in the RPE layer is of significantly lower magnitude and reaches a plateau almost simultaneously with the change observed in the OS-2 layer. The black curve shows the baseline (dark recording) response of the PR OS-2 layer.

Figure 7(c) shows intensity changes in the nerve fiber layer (NFL) (red curve), the IPL (blue curve), and the OPL (green curve). The NFL shows the highest change in intensity amongst these retinal layers, with a maximum change of ∼18% plateauing at ∼1.5  s after the stimulus onset. The IPL and the OPL show an intensity change of ∼5% and ∼10%, respectively, ∼1  s after the stimulus onset. The black curve shows the baseline (dark recording) response of the IPL layer.

Figure 7(d) shows a representative single-flash ERG trace acquired for a stimulus intensity of $3.4\;\mathrm{cd.}s/m^{2}$. The negative a-wave peaks at ∼18  ms after the flash onset, whereas the positive b-wave peaks at ∼35  ms after the flash onset. The gray rectangle in the plot shows the timing and the duration of the white light, single flash stimulus.

Figure 7(e) shows the representative RBF traces from dark recordings (no flash, black curve) and a single flash recording with intensity of $43.4\;\mathrm{cd.}.s/m^{2}$ (red curve). The black curve in the figure shows the blood flow response in the absence of visual stimulation. On average, an RBF change of <20% is present in the absence of visual stimulation. However, a change of as high as 200% is observed with a single flash of $43.4\text{-}\mathrm{cd.}s/m^{2}$ intensity (red curve), which peaked at ∼2  s after the flash onset. The RBF changes are transient, and the flow returns to baseline within ∼5  s from the stimulus onset.

Figure 7(f) shows the representative changes in retinal blood vessel diameter in response to a single-flash stimulus of $43.4\text{-}\mathrm{cd.}s/m^{2}$ intensity. The black curve shows the response in the absence of stimulation, where a slight pulsatile behavior is observed. However, with the onset of stimulation, the vessel dilates to ∼4.5% reaching a maximum value at ∼3  s after the onset of the flash (red curve).

**Fig. 8:**
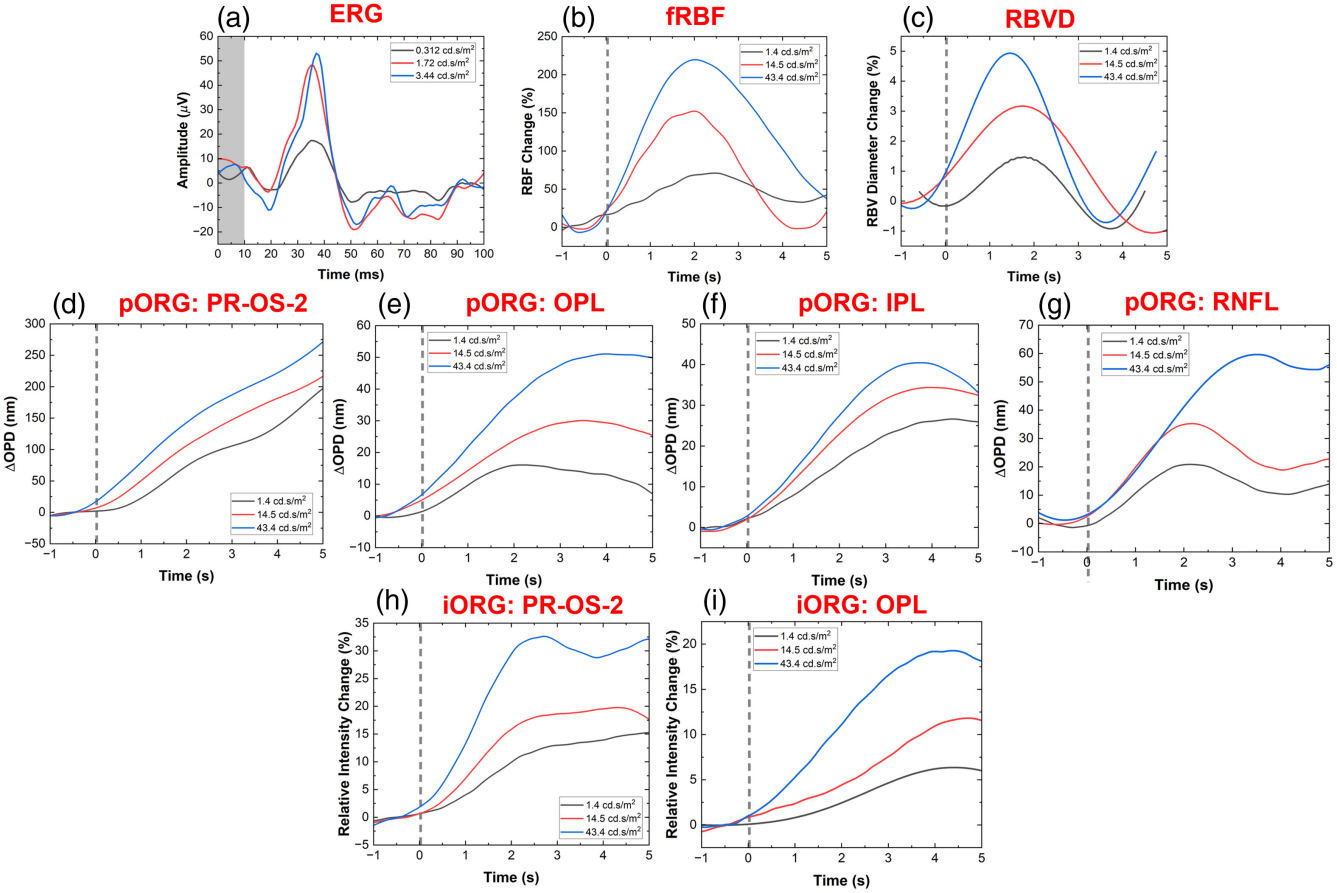
Different responses at different intensities of single-flash stimuli. (a) ERG traces. (b) RBF changes. (c) BVD changes. (d) and (h) Optical path length and intensity changes in the photoreceptor OS-2 layer. (e) and (i) Optical path length and intensity changes in the outer plexiform layer. (f) Optical path length changes in the inner plexiform layer. (g) Optical path length changes in the retinal nerve fiber layer.

Next, we explored the dependence of the retinal response to visual stimulation of different intensities by varying the intensity of the 4-ms single white-light flash stepwise in the range 0 to $43.4\;\mathrm{cd.}s/m^{2}$. Figure 8 shows the representative results for three stimulus intensity values: 1.4, 14.5, and $43.4\;\mathrm{cd.}s/m^{2}$. The ERG recordings [Fig. 8(a)] showed a progressive increase in the amplitude of the a-wave and b-wave, as well as small changes in the peak latency of the b-wave.

The retinal blood flow [Fig. 8(b)] showed a significant increase in the peak magnitude of the transient blood flow changes with progressive increase in the stimulus intensity. The peak latency decreased initially between recordings with 1.4 and $14.5\;\mathrm{cd.}s/m^{2}$ stimulus intensities, and plateaued for flash intensity of $43.4\;\mathrm{cd.}s/m^{2}$. Similar non-linear behavior of the RBF latency was observed by our research group in retinal veins for measurements conducted at the periphery of the optic nerve head in a different study.^67^ The recovery time of the RBF baseline is also affected by the intensity of the visual stimulus and is longer for the highest intensity of the flash. The retinal blood vessel diameter (RBVD) [Fig. 8(c)] showed similar behavior to the RBF. The peak magnitude of the vasodilation increased progressively while the peak latency decreased with the increase of the stimulus intensity.

Figures 8(d) and 8(h) show the phase-based OPD and the intensity-based ORG responses for the photoreceptor OS-2 layer, respectively. The OPD traces showed almost linear increase over the 5-s time with the onset of the flash, and the response was proportional to the increase of the flash intensity, reaching a peak magnitude of ∼300  nm for the strongest flash. Although the magnitude of the intensity-based ORG responses also scaled with the increase of the flash intensity, it plateaued at ∼2.5  s relative to the flash onset, reaching a peak magnitude of ∼30% relative to the baseline (dark recording).

Figures 8(e) and 8(i) show the phase-based (OPD) responses and intensity-based ORG responses measured from the retinal OPL, respectively. The optical path length increase in this layer is almost 10 times smaller in magnitude compared with the response measured from the photoreceptor OS-2 layer, reaching peak magnitudes of ∼20, 30, and 50 nm for the three flash intensity values. The peak latency relative to the onset of the flash also increased with the intensity of the flash in the range of 2 to 4 s. For the lower intensity flashes, the response showed a tendency for return to baseline, whereas the response to the strongest flash ($43.4\;\mathrm{cd.}s/m^{2}$) plateaued after 4 s relative to the flash onset. The intensity-based responses measured for the lower intensity flashes showed transient responses of peak magnitudes of ∼5% and ∼12% and peak latencies of ∼1 and ∼2  s, respectively. The response to the strongest flash plateaued at ∼20% and ∼3.5  s latency relative to the flash onset.

Figure 8(f) shows the phase-based (OPD) responses measured from the retinal IPL. The OPD traces showed a progressive increase of the peak magnitude from ∼20 to ∼40  nm with an increase in the flash intensity, whereas the peak latency decreased from ∼4.3 to ∼3.5  s, respectively. All of the traces showed a tendency to return to baseline.

Figure 8(g) shows the phase-based (OPD) responses measured from the retinal NFL. For the lowest stimulus intensity of $1.4\;\mathrm{cd.}s/m^{2}$, the phase-based ORG response plateaued at ∼20  nm, with ∼2  s peak latency. The OPD response to the stimulus intensity of $14.5\;\mathrm{cd.}s/m^{2}$ peaked at ∼30  nm with ∼2-s peak latency. The strongest flash resulted in almost linear OPD change that peaked at ∼60  nm with ∼3.5-s peak latency.

## 4 Discussion

The novel OCT+ERG modality (imaging technology and image processing algorithms) presented here allows for simultaneous high-resolution imaging of the human retina morphology and probing of the retinal neuronal and blood flow responses to visual stimulation. The OCT system is designed to operate in the same wavelength region (∼800  nm) as commercial OCT systems and has a compact fiber-optic design. The high axial resolution (1.7  μm) is sufficient to resolve the multi-layered structure of the posterior human retina [Fig. 3(a)], including the RPE and the Bruch’s membrane, as well as the triple sub-bands of the inner plexiform layer [Fig. 5(b), yellow arrow] that have been observed in the past with research-grade, visible light (VIS-OCT) technology of similar axial resolution.^27^ The system’s lateral resolution is sufficient to resolve reflections from the tips of individual cone photoreceptors in the healthy human retina located a few degrees away from the fovea [white dots in Figs. 5(e) and 5(f)], as well as the fine capillary network of the outer plexiform layer [Fig. 5(c)].

Integration of the OCT system with a commercial ERG technology serves several purposes:

1. synchronous ORG and ERG recording that allow clinicians access to retinal morphological, functional, and vascular data that are acquired simultaneously
2. easy, flexible control of the visual stimulus and utilization of clinically approved ERG protocols for retinal stimulation.

As the ORG research field is still relatively new, simultaneously recorded ERG traces can serve as a “gold standard” to validate the ORG recordings. Note that although the current design of the visual stimulator allows for flexibility of choice in terms of timing, duration, intensity, frequency pattern, and color of the visual stimulus, it can only generate a wide-field, Maxwellian view illumination of the retina. It also does not provide a fixation pattern for the imaged eye. Future designs of the visual stimulator will include fixation targets for the imaged eye, as well as the ability to project spatial patterns of different sizes on the surface of the retina.

Results from this pilot study demonstrate that the OCT technology and image processing algorithms allow for measurement of both phase-based OPD changes and intensity-based changes from all major retinal layers, as well as transient changes in the retinal blood flow caused by visual stimulation of the retina. The simultaneous measurement of both neuronal responses and transient changes in the blood flow and vasodilation of local blood vessels allows for conducing of non-invasive studies of neurovascular coupling in the human retina. Data from this pilot study show that, in general, both the phase-based OPD and intensity-based responses from retinal layers populated by different types of retinal neurons, to visual stimulation increase in magnitude relative to the baseline (dark recordings) with increase of the stimulus intensity, whereas the latency of the peak responses decreases or increases with increase of the stimulus intensity depending on the retinal layer type.

The largest phase-based OPD changes were observed in the OS-2 sublayer of photoreceptors, which is consistent with the results of rod response-dominated animal and human studies and can be explained by the transient water influx following the phototransduction process of rhodopsin located in the outer segments of photoreceptors.^68^^,^^69^ The OS-2 layer also shows the largest intensity change (∼30%) which can be associated with different factors:

1. Temporary realignment of electrical dipoles within the double lipid membranes that form the OS layer, during the phototransduction process, which results in transient changes of the local refractive index.
2. Conformational changes in the opsin protein can alter the scattering profile of the rhodopsin molecules in the OS layer.
3. Water influx can cause transient changes in the reflectivity profile of the OS layer.

Phase-based OPD and intensity-based changes of almost 10× smaller magnitude were observed from the outer and inner plexiform layers of the human retina in response to the visual stimulation (Figs. 7 and 8). As these retinal layers contain dense capillary networks, these changes are most likely related to a transient increase in the retinal blood flow (retinal blood cells scatter light strongly) and vasodilation of the retinal capillaries. In addition, the inner plexiform layer consists of axons of different types of bipolar cells and dendrites of different types of retinal ganglion cells, activation of which leads to changes in the optical path length of the layer. These results generally agree with the existing study on optical path length difference in the inner plexiform layer by Pfaffle et al.^52^

The retinal NFL is composed of unmyelinated axons of the ganglion cells that conduct action potentials to transmit information from the retina to the brain. The phase-based OPD changes measured from the NFL [Fig. 8(g)] in response to the visual stimulation are most likely due to transient swelling of Müller glial cells, which are located in-between NFL fibers.

The visual stimulation of the human retina caused transient changes both in the local blood flow and the vasodilation of the retinal blood vessels [Figs. 8(b) and 8(c), respectively]. These changes can be explained by the increased metabolic activity of retinal neurons during and immediately after visual stimulation, which increases the demand for oxygen and nutrition delivered by the retinal blood network.

Although we demonstrated that the temporal resolution of the OCT system is sufficient to measure transient changes in the neuronal activity (ORG recordings) and blood vasculature (RBF and RBVD) caused by visual stimulation, 10-s-long recordings pose difficulties for both the imaged subjects (keeping the eye fixated and open) and the image processing algorithms (correction of eye motion artifacts). In this study, subjects tended to blink and lose fixation after ∼7  s, which limited the post-stimulus period to only 5 s and prevented exploration of the recovery process of the ORG and RBF traces back to baseline. Eye motion artifacts arising from involuntary eye motion (heart rate, breathing rate, and ocular muscles twitching) also posed challenges to the filtering process of the ORG and RBF traces and caused the initial increase in the ORG and RBF changes to appear occurring slightly earlier than the stimulus onset. In addition, it is very challenging to image at the “same” location for the imaging protocol mentioned in this study. Small amounts of lateral motion can deem a dataset unusable. This can be addressed by scanning a small region in the slow direction and using this recording as a reference for image registration. Incorporating adaptive optics and pupil tracking into the system can help address these concerns as well. The limited speed of the camera in the current design of the OCT system also prevented measurement of initial fast negative OPD response from the OS-2 layer, corresponding to the ERG a-wave, that was observed by other research groups that used OCT technology with parallel detection such as swept-source OCT,^31^^,^^51^^,^^53^ full-field OCT,^50^ multiple detection channels,^30^ and line-field OCT.^54^^,^^55^

## 5 Conclusion

In summary, we have developed a combined OCT+ERG system for *in vivo*, simultaneous high-resolution imaging of the human retina structure and measurement of fast, transient changes in the retinal neuronal function (ORG and ERG) and the local retinal blood flow (Doppler OCT) induced by visual stimulation. Both optical path length- and intensity-based ORG signals were acquired from the major retinal layers (nerve fiber layer, plexiform layers, photoreceptor inner and outer segments, and the retinal pigmented epithelium). Data acquired in this study provide insight into the neurovascular coupling in the human retina. The OCT+ERG technology and OCT image processing algorithms presented here can serve as valuable research tools in future biomedical and clinical studies of potentially blinding neurodegenerative retinal diseases and brain neurodegenerative conditions that are expressed in the retina.

## Data Availability

The data underlying the results presented in this paper are not publicly available at this time however may be obtained from the corresponding author upon reasonable request.
